# 

**Published:** 1890-11

**Authors:** 


### THE SOUTHERN SURGICAL AND GYNECOLOGICAL ASSOCIATION.

One of the most notable medical conventions that has ever been held in the city of Atlanta will occur on the 11th, 12th and 13th of this month, when the Southern Surgical and Gynecological Association will hold its third annual meeting. Although young in years this body has already become a recognized power in the medical world, and its meetings are looked forward to with great interest by the profession. A glance at the programme, which appears in this number of the JOURNAL, will show that some of the best known members of the profession will participate in the proceedings, and papers of great interest will be presented. Although pre-eminently a Southern Society, as its name indicates, its membership embraces many of the best workers in surgery and gynecology from the Northern States, and some of its most active members are from the other side of Mason and Dixon's line.

The approaching meeting promises to be one of very great interest. The programme already embraces forty papers on a great variety of subjects, and these, with the discussions which will follow them, will furnish a rich feast to all who are interested in surgery or gynecology. The successful meetings already held in Birmingham and in Nashville have demonstrated that an association of this kind fills a long felt want, and the interest manifested upon those occasions warrants the belief that the Atlanta meeting will be no less attractive.

The meetings of the Association are held with open doors, and the whole profession is invited to be present. We trust that the representation from Atlanta and other parts of the State will be a large one. In addition to the advantages to be derived from hearing valuable papers, and still more valuable discussions, an opportunity will be furnished of meeting with men from other portions of the country, whose names have become household words in surgery and gynecology. Many practical hints may be gathered from the utterances of such men, while their personal presence will lend emphasis and interest to their views, and add weight to their authority.

The meetings will be held in Concordia hall, and the entire three days will be devoted to the reading and discussion of papers. All business is transacted by the Executive Committee in order that no valuable time may be consumed in tiresome routine work. Reduced rates will be given over all roads in the South, and reasonable hotel rates have also been promised. The social features of the meeting will not be neglected, and to all who attend, whether members or not, we promise that Atlanta's proverbial hospitality shall be cordially extended.

It is desirable that the membership of the Association should be increased at this meeting, and it is hoped that all who are in sympathy with its work, and who are interested in this special department of medicine, which it covers, will present their names to the Executive Committee for admission.

